# Early Liver Function Parameters Predict Independent Walking Ability After Living Donor Liver Transplantation

**DOI:** 10.3390/medicina61091524

**Published:** 2025-08-25

**Authors:** Satoru Kodama, Takeshi Miyamoto

**Affiliations:** 1 Department of Medical Technology, Division of Rehabilitation Technology, Kumamoto University Hospital, Kumamoto 860-8556, Japan; 2 Department of Physical Medicine and Rehabilitation, Kumamoto University Hospital, Kumamoto 860-8556, Japan; miyamoto.takeshi@kuh.kumamoto-u.ac.jp

**Keywords:** living donor liver transplantation, liver function biomarkers, independent walking ability, predictive modeling, rehabilitation outcomes

## Abstract

*Background and Objectives*: Postoperative physical recovery, particularly the acquisition of independent ambulation, is a critical milestone in rehabilitation following living donor liver transplantation (LDLT). Although liver function markers are conventionally used to assess hepatic physiology, emerging evidence has suggested their potential role as prognostic indicators of physical performance. *Materials and Methods*: This study investigated the association between liver function parameters at the initiation of postoperative physical therapy (total bilirubin [T-Bil], aspartate aminotransferase [AST], and alanine aminotransferase [ALT]) and the independent walking ability of 63 patients who underwent LDLT. A logistic regression model was constructed using these variables, and a receiver-operating characteristic (ROC) curve analysis was performed to evaluate its discriminative performance. Predicted probabilities of each patient were calculated, and the optimal cutoff value was determined based on the Youden Index. *Results*: The multivariate logistic regression model demonstrated a statistically significant association between liver function markers and the ambulation status of a cohort of 63 patients. The ROC curve analysis yielded an area under the ROC curve (AUC) of 0.8416 (95% confidence interval [CI]: 0.715–0.968), indicating strong predictive performance. The optimal cutoff value was 0.865, with sensitivity and specificity of 74.1% and 88.9%, respectively. The bootstrap CI for sensitivity at this threshold ranged from 0.6111 to 0.8519. The Hosmer–Lemeshow test indicated good model fit (*p* = 0.363), and the correct classification rate was 87.3%. *Conclusions*: Liver function test results may be indicators of hepatic dysfunction as well as functional biomarkers that could predict ambulatory outcomes following LDLT. This predictive model may enhance early clinical decision-making regarding rehabilitation and discharge planning. Future prospective studies should be performed to validate the generalizability of these results to broader clinical contexts.

## 1. Introduction

Liver transplantation (LT) is the only definitive treatment for end-stage liver disease (ESLD). Successful LT markedly improves patient survival and quality of life (QOL). However, various factors influence postoperative prognosis, and the importance of skeletal muscle mass (SMM) has received considerable attention recently. Sarcopenia, defined as a reduction in SMM, is now widely recognized as an independent prognostic indicator for LT candidates that correlates not only with increased post-transplant mortality during the waiting list period but also with a higher risk of postoperative complications, particularly infections [1].

SMM is a reliable indicator of the nutritional status and can predict several clinical outcomes, including intensive care unit (ICU) length of stay, total hospitalization duration, and number of endotracheal intubation days following LT. Among male patients, SMM also plays a crucial role in predicting survival and discharge destination [2]. Skeletal muscle is the largest organ in non-obese individuals and accounts for approximately 30–40% of total body weight and 75% of whole-body protein. Therefore, changes in muscle mass serve as critical prognostic markers for chronic diseases. Numerous studies have demonstrated associations between reduced SMM and increased severity, complications, and mortality, especially in cases of chronic liver disease (CLD) [3].

Decreased SMM in patients with non-alcoholic fatty liver disease (NAFLD) and liver cirrhosis correlates with impaired liver function, whereas maintenance or gain of muscle mass is associated with improved hepatic function and more favorable clinical outcomes [4,5]. A study that investigated the relationship between sarcopenia and the 6 min walk test (6MWT) results of LT candidates showed that although sarcopenia was not a significant predictor of mortality during the waiting list period, 6MWT performance was an independent indicator, thus highlighting the clinical importance of mobility in determining the liver disease prognosis [6].

Recent studies have identified frailty as an independent predictor of poor outcomes following living donor LT (LDLT). Frailty is strongly linked to increased postoperative mortality and a higher incidence of complications [7,8,9,10,11]. Preoperative frailty assessments—such as the Liver Frailty Index and Short Physical Performance Battery—are significantly associated with survival rates, length of stay in the ICU, postoperative complication rates, and decline of the physical QOL at 1 year after LDLT [10,12,13,14].

Frailty and sarcopenia increase the mortality risk of patients awaiting LT and contribute to unfavorable outcomes following LDLT. Therefore, transplantation during the mild to moderate stages of frailty is recommended [11,15]. Because frailty is dynamic and potentially reversible, prehabilitation—including nutritional support, exercise therapy, and psychosocial interventions—has been proposed as a method of promoting recovery and enhancing outcomes [8,15,16]. Although frailty tends to worsen transiently at 3 months post-transplantation, partial recovery is often observed by month 12; however, the condition of fewer than 40% of patients recovers to one that is non-frail [12,14].

These findings emphasize the importance of preoperative frailty evaluations and early intervention strategies to improving functional recovery and overall survival following LT [8,10,12,14,16].

Previously, we reported that measurements of psoas muscle mass during the first month of rehabilitation after LDLT were reliable predictors of the independent walking ability [17]. Moreover, recent studies have highlighted a close interrelationship between liver function, SMM, and the acquisition of independent ambulation [18].

Despite the growing recognition of frailty as a determinant of LDLT outcomes, few previous studies have examined this topic in an integrated manner and comprehensively examined the potential to predict ambulation using liver function biomarkers [19]. Key hepatic indicators—such as total bilirubin (T-Bil), aspartate aminotransferase (AST), alanine aminotransferase (ALT), and international normalized ratio (INR)—have not yet been analyzed in an integrative manner in relation to postoperative functional recovery.

This study aimed to address this gap by evaluating the ability of these biochemical markers to predict the achievement of independent walking at the completion of postoperative physical therapy following LDLT. Specifically, whether these markers have statistically significant and independent explanatory power in predictive models based on calculated ambulation probabilities is unclear.

Therefore, this study investigated the association between early postoperative liver function parameters and the acquisition of independent ambulation following LDLT to establish a predictive framework for postoperative recovery. To achieve this, a logistic regression model that incorporated liver function markers that are measurable during the early postoperative phase as explanatory variables was constructed. Predicted probabilities were calculated based on regression coefficients to create a patient-specific quantitative framework to evaluate the ambulation likelihood. Building on these considerations, the discriminative ability of the model was assessed using a receiver-operating characteristic (ROC) curve analysis, and the optimal cutoff point was determined based on the Youden Index. Through this process, the clinical utility of the model was validated as a decision support tool that allows early identification of patients at risk for poor functional recovery. Furthermore, this study explored whether liver function parameters measured at the initiation of physical therapy could reliably predict the achievement of independent walking.

## 2. Materials and Methods

### 2.1. Patient Parameters

Following the approach described by Kodama et al., 2022 [17], we conducted a retrospective cohort study including 63 patients who underwent LDLT for severe hepatic dysfunction (Child–Pugh class C) at our hospital’s Department of Transplant Surgery between January 2010 and April 2024. All participants received physiotherapy during hospitalization. Patients were excluded for the following reasons: they discontinued physiotherapy or died as a result of unstable health conditions, severe infection, fever, or other complications; had a psychiatric illness; were diagnosed with dementia and unable to comply with instructions; experienced mobility restrictions attributable to cardiopulmonary issues or pain; required a second transplantation; lacked a postoperative physiotherapy request; or had insufficient data for analysis.

We evaluated the following 16 clinical factors: age; sex; body mass index (BMI); graft-to-recipient weight ratio (GRWR); model for end-stage liver disease (MELD) score; physiotherapy duration; time from transplantation to physiotherapy initiation; participation in preoperative physiotherapy; length of the ICU stay; underlying renal, respiratory, or cardiac comorbidities; incidence of rejection; postoperative complications; history of diabetes; mobility functional independence measure (FIM) score at the start of physiotherapy; mobility FIM score at the completion of physiotherapy; and change in the FIM score between the start and end of physiotherapy. Additionally, we assessed the following biochemical markers at physiotherapy completion: albumin (Alb); creatinine (Cre); T-Bil; PT (prothrombin time)/INR; sodium (Na); C-reactive protein (CRP); CRP-to-albumin ratio (CAR); platelets (PLT); AST; and ALT. Physical function was measured using mobility-related components of the FIM, including walking, wheelchair use, and stair climbing, at the time of hospital discharge. To assess recovery, patients were categorized based on their mobility FIM score, with a threshold of ≥6 points indicating the ability to walk independently, irrespective of walking speed [20,21].

### 2.2. Ethical Considerations

This study adhered to the principles of the Declaration of Helsinki and Ethical Guidelines for Medical and Health Research Involving Human Subjects. All parameters and test results examined were part of routine clinical practice; therefore, the individuals could not be identified. Additionally, we obtained approval from the Ethics Committee of Kumamoto University Hospital (Ethics No. 3208) to use the data for analytical research. Participants were recruited for this study by posting a request for cooperation in clinical research on our hospital website, and an opt-out option was provided.

### 2.3. Statistical Analysis

Patients were divided into the following two groups based on their mobility FIM score at discharge from physiotherapy: those who could walk independently (FIM score ≥ 6) and those who could not. We used the Shapiro–Wilk test to compare these groups based on data from several factors, including the biochemical markers. Fisher’s exact test was used for categorical variables such as sex, diabetes history, underlying diseases, rejection incidence, postoperative complications, and pre-LDLT physiotherapy. We used the Mann–Whitney *U* test for continuous variables (age, BMI, GRWR, MELD score, physiotherapy duration, time from LDLT to the start of physiotherapy, length of the ICU stay, Alb, Cre, T-Bil, PT/INR, Na, PLT, CRP, CAR, AST, ALT, mobility FIM score at the start of physiotherapy, mobility FIM score at the completion of physiotherapy, change in the FIM score between the start and end of physiotherapy). Then, we used Spearman’s rank correlation coefficient to explore the correlation between mobility and various factors, including age, sex, BMI, GRWR, MELD score, physiotherapy duration, time from LDLT to the start of physiotherapy, length of the ICU stay, mobility FIM score at the start of physiotherapy, change in the FIM score between the start and end of physiotherapy, and biochemical markers. In addition to variables that reached statistical significance in the univariate analysis (*p* < 0.05), clinically relevant factors with potential confounding effects were included in the multivariate logistic regression model to enhance interpretability and validity. To identify factors associated with achieving independent walking at discharge, we conducted a multiple logistic regression analysis. To construct the predictive model, a binary logistic regression analysis was performed using SPSS Statistics version 2.0 (IBM Corp., Chicago, IL, USA). The dependent variable was the attainment of independent ambulation (1 = independent walking; 0 = non-independent). The explanatory variables included liver function markers (T-Bil, AST, ALT). After model development, the [Save] function was used to generate the predicted probability for each case (PRE_1), which was subsequently evaluated using ROC curve analysis. The goodness-of-fit of the logistic regression model was assessed using the Hosmer–Lemeshow test. A non-significant result (*p* > 0.05) indicated that the model was well-calibrated and demonstrated adequate fit to the observed data.

In the ROC analysis, the predicted probability was designated as the test variable, and the actual ambulation status (independent walking or non-independent walking) served as the categorical variable. The area under the ROC curve (AUC) and the optimal cutoff value were determined. The cutoff was calculated based on the Youden Index, which maximizes the sum of sensitivity and specificity. Furthermore, model validation was performed by evaluating its robustness through bootstrap resampling. All statistical analyses were performed using IBM SPSS Statistics version 20.0 (IBM Corp.) and R (version 2.5; R Foundation for Statistical Computing, Vienna, Austria).

## 3. Results

This study included 63 patients (27 male and 36 female patients) with a mean age of 60.0 years (standard deviation, ±11.8 years). The independent walking group included 54 patients, whereas the non-independent walking group included 9 patients.

Basic characteristics included hepatitis C cirrhosis (nine cases), alcoholic cirrhosis (17 cases), cryptogenic cirrhosis (11 cases), hepatitis cirrhosis (three cases), acute liver failure (seven cases), primary sclerosing cholangitis (three cases), biliary atresia (one case), familial amyloid polyneuropathy (two cases), non-alcoholic steatohepatitis (six cases), multiple hepatic cysts (two cases), and primary biliary cholangitis (two cases) (Table 1).

Underlying diseases included renal disease (16 patients), respiratory disease (11 patients), and cardiac disease (six patients) (Table 2). Based on the Clavien–Dindo classification, the major complications observed during postoperative hospitalization were as follows: grade 4a complications in one patient (pleural effusion); grade 3b complications in 11 patients (including intraabdominal hemorrhage in six patients, intrathoracic hemorrhage in two patients, and abdominal wall scar hernia in three patients); and grade 3a complications in 14 patients (including venous thrombosis, pleural effusion, biliary fistula, and bile duct anastomotic leakage) (Table 3). All patients were classified as Child–Pugh class C, indicating severe hepatic dysfunction.

Spearman’s rank correlation analysis did not reveal any statistically significant associations among the examined variables (Table 4 and Table 5). Although some individual parameters showed notable differences, most variables did not demonstrate consistent statistical significance, suggesting an overall lack of strong associations within the dataset (Table 4 and Table 5). The Relationships between liver function markers, patient characteristics, and FIM scores are described in Table S1.

In the logistic univariate regression analysis, a statistically significant association was observed between the acquisition of independent ambulation at the completion of physical therapy and the change in FIM scores during the rehabilitation period (Table 5).

**Figure 1:**
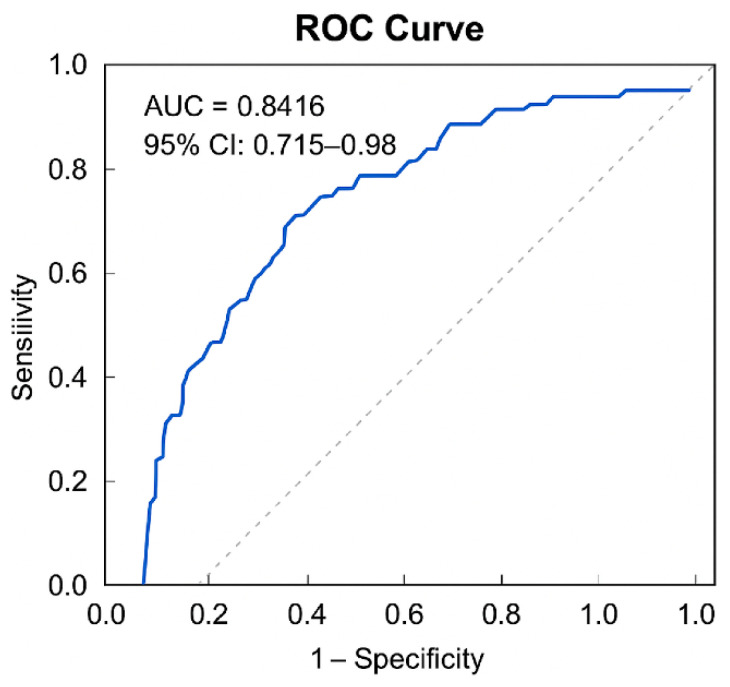
Receiver-operating characteristic (ROC) curve for predictive model performance. The ROC curve illustrates the diagnostic discrimination of the model. The vertical axis shows sensitivity (true positive rate), whereas the horizontal axis represents 1—specificity (false positive rate). The blue line indicates the performance of the model, and the diagonal dashed line denotes random classification (AUC = 0.5). The model yielded an area under the ROC curve (AUC) of 0.8416 with a 95% confidence interval of 0.715–0.98, suggesting strong discriminatory capability.

The ROC curve analysis was conducted using predicted probabilities derived from the logistic regression model that incorporated liver function markers (T-Bil, AST, ALT) as explanatory variables. The AUC was 0.842, demonstrating high discriminative ability (*p* = 0.001) (Figure 1). The 95% confidence interval (CI) was 0.715 to 0.968.

Based on the maximum Youden Index, the optimal cutoff value of the predicted probability was calculated as 0.865. At this threshold, sensitivity and specificity were 74.1% and 88.9%, respectively (Table 6). The Hosmer–Lemeshow test confirmed good model fit (*p* = 0.363), and the correct classification rate was 87.3%.

The receiver-operating characteristic (ROC) curve analysis was performed to evaluate the discriminative ability of the predictive model for independent ambulation. The area under the ROC curve (AUC) was 0.8416, indicating excellent discriminative performance. The 95% confidence interval (CI) was 0.715 to 0.968.

Based on the maximum Youden Index, the optimal cutoff value was 0.865. At this threshold, the model demonstrated a sensitivity of 0.741 and a specificity of 0.889. The corresponding Youden Index was 0.630.

Furthermore, model robustness was verified through bootstrap resampling. The bootstrap CI for sensitivity at the optimal cutoff ranged from 0.6111 to 0.8519, supporting the reliability and stability of the predictive performance (Table 7). These findings indicate that the logistic regression model that incorporated liver function parameters provides meaningful clinical utility for predicting the attainment of independent ambulation following LDLT (Table 6 and Table 7; Figure 1).

## 4. Discussion

This study investigated the association between liver function parameters (T-Bil, AST, and ALT) and independent ambulation status in patients following LDLT. In the univariate logistic regression analysis, none of these variables reached statistical significance. However, in the multivariate logistic regression model, T-Bil emerged as a significant predictor of ambulation status (*p* = 0.04; 95% CI: 1.02–1.86). This suggests that T-Bil may independently contribute to postoperative functional outcomes when adjusted for other clinical variables (Table 5). This finding aligns with previous literature reporting associations between liver function and motor or muscular performance. T-Bil reflects the hepatic metabolism and biliary excretion of bilirubin, a breakdown product of erythrocytes. Elevated T-Bil levels are indicative of hepatic dysfunction and have been shown to negatively impact exercise tolerance. Notably, in LDLT recipients undergoing the 6MWT, higher T-Bil levels were negatively correlated with walking distance [22].

Nonetheless, T-Bil alone did not demonstrate a strong correlation with independent ambulation, indicating limited predictive accuracy when used in isolation. Therefore, we constructed a multivariate logistic regression model incorporating additional hepatic enzymes, including AST and ALT, alongside T-Bil. This composite model yielded statistically significant predictive performance. Furthermore, the optimal threshold for predicted probability was calculated to be 0.8416, demonstrating adequate discriminative power in identifying ambulation status. These results suggest that a mathematical model incorporating T-Bil may serve as a clinically applicable tool for the early detection of postoperative functional decline. Such a model could support the optimization of physical therapy initiation and discharge planning and may represent a novel indicator for predicting functional prognosis in the postoperative setting. Its implementation in clinical practice has the potential to enhance decision-making and improve patient outcomes.

Evidence regarding heart failure further underscores the clinical relevance of hepatic function in relation to physical capacity [23]. Hanada et al. similarly demonstrated a negative correlation between peak oxygen uptake (VO_2_ peak) during cardiopulmonary exercise testing and hepatic enzymes such as T-Bil and AST, suggesting that impaired liver function may contribute to reduced exercise tolerance [24].

The MELD-XI score, which is based on T-Bil and Cre, has been conventionally used for organ allocation associated with LT, but it has also gained traction as a predictor of physical function for populations with heart failure. Noda et al. revealed that the MELD-XI score outperformed BNP when used to predict handgrip strength, lower extremity power, gait speed, and 6MWT distance; therefore, it has been identified as a robust marker of muscle wasting and functional decline [25]. These findings align closely with the results of the current study and further support the correlation between hepatic function and physical function.

Although traditionally regarded as markers of hepatocellular injury, AST and ALT have been recently recognized for their expression in skeletal muscle and cardiac muscle; therefore, they have been implicated in systemic disorders, including sarcopenia. Ruhl and Everhart found a positive correlation between elevated ALT/AST levels and cardiovascular mortality [26]. Yang et al. also linked elevated ALT levels with increased all-cause mortality among patients with diabetes [27].

In the present study, ALT was identified as a statistically significant predictor of independent ambulation in the multivariate logistic regression model. However, the odds ratio for ALT was accompanied by a narrow 95% CI (1.00–1.07), with the lower bound precisely at 1.00, which warrants cautious interpretation of this result.

The proximity of the lower bound to 1.00 suggests that, although the association reached statistical significance, its clinical impact may be limited. In other words, the effect size of ALT appears small, and its influence on ambulation outcomes may not be sufficiently robust to inform clinical decision-making with confidence.

Moreover, the narrow CI may reflect statistical uncertainty arising from the limited number of non-events in the dataset. Specifically, the non-ambulatory group comprised only nine cases, which may have introduced instability in the estimation of continuous variables such as ALT. It is important to note that a narrow CI does not necessarily indicate high precision, particularly when the sample size is small or the data distribution is skewed. Under such conditions, the interval may instead reflect underlying uncertainty in the model estimates.

Taken together, although ALT demonstrated statistical significance in the multivariate model, its interpretation should be approached with caution and regarded as preliminary. Further studies with larger sample sizes are warranted to validate the predictive value and clinical relevance of ALT in this context.

We constructed a logistic regression model to predict the acquisition of independent ambulation using early postoperative liver function indicators as explanatory variables. The results of the univariate logistic regression analysis demonstrated a statistically significant association between independent ambulation at the completion of physical therapy and the change in FIM scores during the rehabilitation period. However, the change in FIM scores was excluded from the multivariate analysis based on the following methodological considerations. First, the FIM score includes items that directly assess ambulation, and the improvement in FIM scores may structurally overlap with the outcome of interest, specifically, the acquisition of independent walking. This overlap could compromise the independence between the explanatory and outcome variables and introduce circular causality within the model. Second, the change in FIM scores represents the outcome of a rehabilitative intervention; therefore, it should be regarded as a post-treatment measure rather than a predictive variable in the study design. Incorporating such a retrospective outcome variable as an explanatory factor could disrupt the statistical coherence of the model and complicate causal interpretation. Therefore, we opted to treat the change in FIM scores as a supplementary analytic variable rather than include it in the multivariate model.

Liver function indicators were incorporated in a multivariate logistic regression analysis. The results demonstrated statistically significant associations within the multivariate model. This finding suggests that liver function markers may be influenced by interactions and confounding effects of other clinical variables, and that their true associations may be underestimated when evaluated in isolation. Liver function test results may not only reflect hepatic physiology following LT but also serve as composite indicators of the systemic status, including nutritional condition, immune response, inflammatory activity, and SMM.

We fully acknowledge the clinical and research importance of sarcopenia and frailty indicators, particularly those derived from muscle mass assessments using computed tomography (CT) imaging. Numerous recent studies have demonstrated that imaging-based metrics—such as the muscle index calculated from cross-sectional muscle area at the lumbar vertebral level—are strongly associated with postoperative recovery and functional outcomes. These indicators are widely recognized as valuable biomarkers reflecting a patient’s overall physical status and physiological reserve, especially in older adults and individuals with chronic conditions.

However, in the present study, we were unable to incorporate these variables into our analysis. The primary limitations were related to postoperative imaging protocols and the difficulty of obtaining standardized data for quantitative muscle mass evaluation from CT images. Specifically, during the acute postoperative phase, clinical priorities are directed toward monitoring organ function and managing complications, and CT scans are not routinely performed for the purpose of muscle assessment. Consequently, the timing and anatomical regions of imaging are often inconsistent and lack standardization, making reliable measurement of muscle mass challenging.

Given these constraints, we excluded sarcopenia and frailty indicators from our analysis. Nonetheless, we recognize that these metrics may serve as independent predictors of postoperative functional recovery and believe they should be actively incorporated into future research. In particular, standardizing quantitative muscle mass assessment using pre- and postoperative CT imaging and integrating these data with biochemical markers may facilitate the development of more precise and individualized models for predicting functional outcomes.

Therefore, in this study, a predictive model of independent ambulation was developed using a logistic regression analysis that incorporated liver function parameters measurable during the early postoperative period as explanatory variables. By calculating predicted probabilities for each patient, we established a framework to quantitatively assess the likelihood of ambulation acquisition on an individual basis. Furthermore, ROC curve analysis was used to evaluate the discriminative performance of the model, and the optimal cutoff point was determined based on the maximum Youden Index. This approach enabled validation of the model’s clinical utility as a decision support tool that could allow early identification of patients at higher risk for delayed ambulation. The resulting model demonstrated a strong predictive capability (AUC of 0.8416). The optimal cutoff value was 0.865 (sensitivity, 0.741; specificity, 0.889). The corresponding Youden Index was calculated as 0.630.

These findings suggest that the predictive model that incorporated liver function markers had a clinically meaningful ability to predict the attainment of independent ambulation. This highlights the potential for liver function test results to be redefined not only as indicators of organ-specific damage but also as functional biomarkers that reflect postoperative recovery and long-term physical outcomes. The predictive model developed in this study demonstrated clinical utility from multiple perspectives as a personalized decision support tool that could be used to assess functional recovery after LDLT. The use of liver function biomarkers to evaluate the functional prognosis in the early postoperative phase is considered highly effective. Recovery after LDLT varies substantially among individuals because of differences in systemic metabolic burden, immunological responses, and nutritional status, which influence physical functional improvement. Notably, liver function indicators such as T-Bil, AST, and ALT reflect the physiological performance of the graft, including parenchymal recovery, bile excretory capacity, and coagulation balance. By integrating these parameters, the model enabled the prediction of postoperative independent ambulation potential. This model is an objective tool that can be used to determine the need for physical therapy intervention during the early postoperative phase and may support the formulation of prioritized rehabilitation plans for patients at risk for delayed recovery. Furthermore, the predicted probabilities generated from the logistic regression model in this study were functional indicators of individualized risk. Traditional binary classifications have limited ability to account for interpatient variability of functional outcomes such as ambulation acquisition. In contrast, the use of probability modeling enables stratified classification into categories such as “high likelihood,” “moderate likelihood,” or “low likelihood” for ambulation acquisition. Risk stratification based on probability values is clinically useful for clinical decision-making in terms of prioritizing physical therapy, allocating rehabilitation resources, and planning discharge support strategies. Additionally, sharing these predicted probabilities with patients and families can facilitate communication with healthcare providers and support the setting of realistic recovery goals. The discriminative performance of the model was objectively validated using ROC curve analysis, with the AUC and Youden Index applied as evaluation metrics. The ROC analysis is a standard approach used to assess predictive accuracy, and the AUC value reflects the model’s overall discriminative power. In this study, the AUC for the predicted probabilities was sufficiently high, indicating the model’s validity as a clinical decision support tool. Moreover, the optimal cutoff value derived using the Youden Index was a probability threshold that balances sensitivity and specificity. This threshold may serve as a practical early indicator of the risk of poor ambulatory recovery in the postoperative setting. Therefore, the predictive model in this study can be utilized in multiple dimensions as a clinically valuable tool to support functional outcome assessments after LT, guide individualized rehabilitation strategies, and provide quantitative evidence to supplement clinical decision-making. Its application is expected to enhance the quality of rehabilitation medicine. Despite the relatively small sample size and retrospective nature of the analysis, this approach increased its credibility. The use of early postoperative liver function biomarkers to predict individualized outcomes provides greater clinical applicability compared to that described by previous studies. Moreover, this model has potential as a practical decision support tool. Notably, the evaluation of the discriminative performance using ROC curve analysis and the determination of an optimal cutoff value using the Youden Index are particularly useful in the clinical setting.

In this study, a logistic regression model incorporating liver function markers demonstrated high discriminative ability for predicting the acquisition of independent ambulation after LDLT. The findings suggest that early postoperative blood data may help predict mobility outcomes, thus potentially contributing to individualized rehabilitation planning and efficient allocation of medical resources. Furthermore, the combination of liver function indicators may facilitate stratification of patients at rehabilitation initiation and have clinical implications for estimating the functional prognosis and tailoring intervention intensity.

Although the change in FIM scores was statistically significant according to the univariate analysis, it was excluded from the multivariate model to avoid structural overlap with the outcome variable and prevent circular causality. This decision was made to preserve the conceptual integrity of the model design. Future studies should incorporate more detailed and objective parameters of postoperative functional recovery, such as walking speed, ambulation distance, or stair-climbing performance, to enhance predictive precision.

Moreover, Spearman’s rank correlation analysis did not reveal statistically significant associations among the examined variables. The lack of consistent correlations may be attributable to physiological variability, interindividual differences in postoperative recovery, and temporal mismatches between the measurements of biochemical markers and assessments of functional outcomes.

Nevertheless, some limitations must be acknowledged. First, although the total sample size was 63, the number of patients classified into the non-ambulatory group was extremely small (*n* = 9), which may have substantially reduced the statistical power of the analysis. Statistical power refers to the ability to detect a true difference or association as statistically significant. When power is low, the risk of a Type II error increases—that is, failing to identify a statistically significant result despite the presence of a clinically meaningful effect. Such errors may lead to the erroneous conclusion that no difference or association exists, thereby overlooking potentially important clinical insights. In the present study, the limited number of non-ambulatory cases may have contributed to the lack of statistical significance in group comparisons and ROC analyses, even if a true association was present. Therefore, the results of this study should be interpreted with caution, considering not only statistical significance but also effect sizes and clinical relevance. Future research involving larger sample sizes and multicenter collaborations will be essential to validate and reproduce these findings.

Second, the multivariate logistic regression model used in this study included three predictor variables, whereas the number of non-events—specifically, patients who did not achieve independent ambulation—was limited to nine, as noted above. It is widely recognized that logistic regression models require a minimum of 10 events (or non-events) per predictor variable to ensure model stability and reliability. This rule of thumb serves as a statistical guideline to reduce the risk of overfitting, which occurs when a model is overly tailored to the training data as a result of insufficient sample size. Overfitting may result in high predictive accuracy within the original dataset but poor generalizability to external populations. In other words, the model may not perform reliably when applied to new patient groups, thereby limiting its clinical applicability. Given that our model incorporated three predictors with only nine non-events, the ratio of variables to events was imbalanced, and we recognize that this may have increased the risk of overfitting. Consequently, the cutoff values and predictive performance derived from this model may not be reproducible in other institutions or patient populations.

In light of these limitations, caution is warranted when applying this model to external cohorts. Ideally, future studies should involve larger sample sizes and prospective multicenter designs to allow for model reconstruction and external validation, thereby confirming the robustness and generalizability of the identified predictors.

Third, the limited sample size of the non-independent ambulation group imposed restrictions on the number of variables that could be included in the multivariate analysis. Although the change in FIM scores was statistically significant, it was intentionally excluded because of the aforementioned reasons. Fourth, the retrospective design and single-center setting may have introduced bias attributable to institutional characteristics, case selection practices, and evaluation environments, which cannot be fully excluded. Fifth, liver function markers were obtained at a single time point during the early postoperative phase, thus limiting the ability to capture dynamic physiological changes or longitudinal progression. In addition, the assessment of independent ambulation was based on clinical records; therefore, accounting for non-medical factors such as assistive device use or environmental conditions that may influence mobility outcomes was difficult.

Despite these limitations, the present study provides valuable preliminary insights regarding the potential relationship between liver function indicators and postoperative ambulation capacity after LDLT. These findings highlight a promising direction for the development of new functional assessment tools for rehabilitation medicine. Further refinement and validation of the proposed model, including integration with electronic medical record systems to allow real-time risk visualization and clinical implementation of predictive algorithms, may lead to the establishment of individualized prognostic tools for postoperative care.

## 5. Conclusions

Early postoperative liver function markers, including T-Bil, AST, and ALT, were significantly associated with independent ambulation after LDLT. The predictive model exhibited strong performance and may allow early identification of patients at risk for delayed physical recovery, thus supporting clinical decisions regarding rehabilitation and discharge planning.

## Figures and Tables

**Table 1:** Basic characteristics.

	Age (Years)	Sex (M/F)	BMI (kg/m^2^)	Underlying Diseases
**Independent Walking Group**				
Patient 1	45	F	33.8	Alcoholic cirrhosis
Patient 2	60	M	25.4	Cryptogenic cirrhosis
Patient 3	21	F	34.5	Biliary atresia
Patient 4	51	M	24.4	Hepatitis C cirrhosis
Patient 5	57	F	21.0	Alcoholic cirrhosis
Patient 6	57	M	27.7	Cryptogenic cirrhosis
Patient 7	54	M	19.2	Alcoholic cirrhosis
Patient 8	35	M	24.4	Alcoholic cirrhosis
Patient 9	51	F	14.8	FAP
Patient 10	53	M	25.3	Hepatitis C cirrhosis
Patient 11	39	M	19.0	Alcoholic cirrhosis
Patient 12	60	F	21.6	Cryptogenic cirrhosis
Patient 13	62	M	26.2	Acute liver failure
Patient 14	54	M	25.6	Hepatitis B cirrhosis
Patient 15	65	F	27.0	Alcoholic cirrhosis
Patient 16	54	F	16.8	Acute liver failure
Patient 17	67	F	23.7	Hepatitis B cirrhosis
Patient 18	52	F	29.1	Cryptogenic cirrhosis
Patient 19	23	F	18.6	Primary sclerosing cholangitis
Patient 20	49	F	34.4	Acute liver failure
Patient 21	64	F	24.2	Cryptogenic cirrhosis
Patient 22	42	M	21.4	Alcoholic cirrhosis
Patient 23	56	M	26.0	NASH
Patient 24	33	M	17.3	Acute liver failure
Patient 25	65	F	32.9	NASH
Patient 26	19	M	18.7	Cryptogenic cirrhosis
Patient 27	23	F	23.6	Cryptogenic cirrhosis
Patient 28	51	M	23.9	Alcoholic cirrhosis
Patient 29	52	M	21.1	Alcoholic cirrhosis
Patient 30	54	F	24.1	Hepatitis C cirrhosis
Patient 31	33	M	22.1	Primary sclerosing cholangitis
Patient 32	50	M	23.1	Hepatitis B cirrhosis
Patient 33	51	F	33.0	NASH
Patient 34	48	M	35.8	Alcoholic cirrhosis
Patient 35	53	M	27.8	Hepatitis C cirrhosis
Patient 36	65	F	20.5	Hepatitis C cirrhosis
Patient 37	47	F	15.8	Multiple hepatic cysts
Patient 38	34	F	20.8	Primary sclerosing cholangitis
Patient 39	48	M	29.4	Alcoholic cirrhosis
Patient 40	65	F	16.3	Multiple hepatic cysts
Patient 41	58	M	18.2	Cryptogenic cirrhosis
Patient 42	51	F	23.7	Acute liver failure
Patient 43	61	F	22.5	NASH
Patient 44	56	F	28.0	NASH
Patient 45	55	F	24.6	Acute liver failure
Patient 46	54	F	23.9	Hepatitis C cirrhosis
Patient 47	54	F	18.9	Cryptogenic cirrhosis
Patient 48	58	F	21.8	Primary biliary cholangitis
Patient 49	47	M	21.1	NASH
Patient 50	52	F	25.5	Alcoholic cirrhosis
Patient 51	48	M	25.9	Alcoholic cirrhosis
Patient 52	58	M	24.0	Alcoholic cirrhosis
Patient 53	69	F	20.6	Primary biliary cholangitis
Patient 54	55	F	19.6	Alcoholic cirrhosis
**Non-independent walking group**				
Patient 1	69	F	29.2	Hepatitis C cirrhosis
Patient 2	59	F	25.9	Hepatitis C cirrhosis
Patient 3	63	F	24.7	Cryptogenic cirrhosis
Patient 4	56	F	26.4	Cryptogenic cirrhosis
Patient 5	62	M	25.0	Hepatitis C cirrhosis
Patient 6	37	F	21.4	Acute liver failure
Patient 7	56	M	20.2	FAP
Patient 8	35	M	22.7	Alcoholic cirrhosis
Patient 9	37	F	17.5	Alcoholic cirrhosis

F = female; FAP = fibroblast activation protein; M = male; NASH = nonalcoholic steatohepatitis.

**Table 2:** Incidence of underlying disease.

	Cases
Renal disease	16
Pulmonary diseases	11
Cardiac diseases	6

**Table 3:** Categorization of complications based on the Clavien–Dindo classification (grade III or higher).

Clavien–Dindo Classification	Complication	Cases
IVa	Pleural effusion	1
IIIb	Intraabdominal hemorrhage	6
	Intrathoracic hemorrhage	2
	Abdominal wall scar hernia	3
IIIa	Venous thrombosis	6
	Pleural effusion	4
	Biliary fistula	3
	Bile duct anastomosis	1

**Table 4:** Comparison of clinical parameters of the independent and non-independent walking groups.

Variable	Total (*n* = 63)	Independent Walking Group (*n* = 54)	Non-Independent Walking Group (*n* = 9)	*p*-Value
Age (years)	60.0 (11.8)	50.7 (11.7)	52.7 (12.9)	0.38
Sex (M/F)	27/36	0.8	0.5	0.54
BMI (kg/m^2^)	23.8 (4.8)	23.9 (5.0)	23.7 (3.6)	0.4
GRWR (%)	0.9 (0.2)	0.9 (0.2)	0.9 (0.2)	0.70
MELD score	16.6 (8.4)	16.3 (7.5)	18.4 (12.9)	0.84
Physical therapy (days)	60.3 (41.8)	57.8 (37.0)	75.3 (64.8)	0.38
Days from LDLT to the start of physical therapy	10.3 (6.0)	10.1 (6.3)	11.8 (3.7)	0.22
Physical therapy before LDLT (present or absent)	0.54 (0.5)	0.56 (0.5)	0.44 (0.53)	0.09
ICU admission days	7.4 (7.3)	7.2 (7.7)	8.4 (5.2)	0.18
Mobility FIM score at the start of physiotherapy	2.1 (0.2)	2.2 (2.0)	1.2 (0.7)	0.2
Change in the FIM score between the start and end of physiotherapy	4.6 (0.3)	4.7 (2.0)	2.0 (1.6)	0.21
History of diabetes (present or absent)	37/26	0.4 (0.5)	0.6 (0.5)	0.35
Underlying disease	27/36	0.5 (0.5)	0.2 (0.4)	0.18
Presence or absence of rejection	9/54	0.2 (0.4)	0.1 (0.3)	0.77
Postoperative complications	35/28	0.6 (0.5)	0.4 (0.5)	0.42
After physical therapy				
Alb (g/dL)	2.8 (0.4)	2.8 (0.3)	2.8 (0.5)	0.65
Cre (g/dL)	1.1 (0.1)	1.2 (1.0)	0.6 (0.3)	0.05 *
T-Bil (g/dL)	6.1 (0.6)	6.5 (5.3)	3.4 (2.5)	0.04 *
PT (INR)	1.4 (0.0)	1.5 (0.4)	1.2 (0.2)	0.1
PLT (10^3^/μL)	10.3 (1.4)	10.5 (12.1)	9.3 (5.4)	0.52
Na (mEq/L)	136.8 (0.5)	136.9 (4.3)	136.3 (3.7)	0.55
CRP (g/dL)	1.5 (0.2)	1.7 (1.5)	0.7 (0.6)	0.07
CAR	0.5 (0.1)	0.6 (0.5)	0.3 (0.3)	0.07
AST (U/L)	71.2 (12.9)	64.9 (58.9)	109.1 (236.8)	0.16
ALT (U/L)	114.2 (14.5)	114.4 (104.0)	109.8 (175.6)	0.12

Alb = albumin; ALT = alanine aminotransferase; AST = aspartate aminotransferase; BMI = body mass index; CAR = CRP-to-albumin ratio; Cre = creatinine; CRP = C-reactive protein; F = female; FIM = functional independence measure; GRWR = graft-to-recipient weight ratio; ICU = intensive care unit; INR = international normalized ratio; LDLT = living donor liver transplantation; M = male; MELD = model for end-stage liver disease; Na = sodium; PLT = platelets; PT = prothrombin time; T-Bil = total bilirubin. * Significant difference *p* < 0.05.

**Table 5:** Factors associated with independent walking at the start of physiotherapy.

FIM Motor Locomotion Walk Score at the Start of Physical Therapy				
	Single Regression Analysis		Multivariate Analysis ^ab^	
Parameter	95% CI	*p*	OR (95% CI)	*p*
Age	−0.03 to 0.04	0.85	-	-
Sex	−0.24 to 0.13	0.54	-	-
BMI	−0.02 to 0.02	0.91	-	-
GRWR	−1.82 to 1.86	0.98	-	-
MELD	−0.06 to 0.03	0.59	-	-
Physical therapy (days)	−0.01 to 0.01	0.99	-	-
Days from LDLT to the start of physical therapy	−0.08 to 0.04	0.48	-	-
Physical therapy before LDLT (present or absent)	−0.62 to 0.87	0.74	-	-
ICU admission days	−0.07 to 0.03	0.36	-	-
History of diabetes (present or absent)	−1.31 to 0.17	0.13	-	-
Underlying disease	−0.43 to 1.06	0.40	-	-
Mobility FIM score at the start of physiotherapy	0.75 to 3.78	0.21	-	-
Change in the FIM score between the start and end of physiotherapy	1.23 to 2.56	0.00^*^	-	-
Presence or absence of rejection	−1.16 to 0.97	0.86	-	-
Postoperative complications	−0.39 to 1.1	0.34	-	-
At the start of physical therapy				
Alb (g/dL)	0.19 to 17.5	0.61	-	-
Cre (g/dL)	0.58 to 153.7	0.12	-	-
T-Bil (g/dL)	0.96 to 1.66	0.1	1.02 to 1.86	0.04 *
PT (INR)	0.55 to 294.4	0.11	-	-
PLT (10^3^/μL)	0.94 to 1.1	0.78	-	-
Na (mEq/L)	0.87 to 1.22	0.73	-	-
CRP (g/dL)	0.87 to 6.3	0.09	-	-
CAR	0.65 to 108.3	0.1	-	-
AST (U/L), mean (SD)	0.1 to 1.0	0.28	0.95 to 1.0	0.02 *
ALT (U/L), mean (SD)	0.99 to 1.0	0.9	1.0 to 1.07	0.04 *

Alb, albumin; ALT, alanine transaminase; AST, aspartate aminotransferase; BMI, body mass index; CAR, CRP-to-albumin ratio; CI, confidence interval; Cre, creatinine; CRP, C-reactive protein; FIM, functional independence measure; GRWR, graft-to-recipient weight ratio; INR, international normalized ratio; LDLT, living donor liver transplantation; MELD, model for end-stage liver disease; Na, sodium; OR, odds ratio; SD, standard deviation; T-Bil, total bilirubin. ^a^ Hosmer–Lemeshow test: (*p* = 0.363). ^b^ Correct classification rate: 87.3%. * Significant difference *p* < 0.05.

**Table 6:** ROC curve-based performance metrics of the predictive model for independent ambulation.

Metric	Value
AUC	0.842
(95% CI)	0.715–0.968
Optimal cutoff value	0.865
Sensitivity	0.741 (74.1%)
Specificity	0.889 (88.9%)
Youden Index	0.63

**Table 7:** Bootstrap confidence interval for sensitivity at optimal threshold.

Threshold	Sensitivity (95% CI)
0.8649	0.7407 (0.6111–0.8519)

CI, confidence interval.

## Data Availability

The original contributions presented in this study are included in the article. Further inquiries can be directed to the corresponding author.

## Supplementary Materials

The following supporting information can be downloaded at: https://www.mdpi.com/article/10.3390/medicina61091524/s1, Table S1: Relationships between liver function markers, patient characteristics, and FIM scores.

## Abbreviations

The following abbreviations are used in this manuscript:

Alb	Albumin
ALT	Alanine transaminase
AST	Aspartate aminotransferase
BMI	Body mass index
CAR	CRP-to-albumin ratio
CLD	Chronic liver disease
Cre	Creatinine
CRP	C-reactive protein
FIM	Functional independence measure
GRWR	Graft-to-recipient weight ratio
ICU	Intensive care unit
INR	International normalized ratio
LDLT	Living donor liver transplantation
MELD	Model for end-stage liver disease
MELD-XI	Model for end-stage liver disease XI
NAFLD	Non-alcoholic fatty liver disease
Na	Sodium
PT	Prothrombin time
QOL	Quality of life
ROC	Receiver-operating characteristic
T-Bil	Total bilirubin
CT	Computed tomography
PLT	Platelets
SMM	Skeletal muscle mass
ESLD	End-stage liver disease
CI	Confidence interval
6MWT	6 min walk test
AUC	Area under the ROC curve
